# Transitions in Sarcopenia Status and Cognitive Trajectories Among Middle-Aged and Older Adults in China: Longitudinal Cohort Study

**DOI:** 10.2196/78277

**Published:** 2025-12-16

**Authors:** Chun Luo, Hao Wu, Xiaoying Shen, Shuang Han, Lv Lin, Bingyang Liu

**Affiliations:** 1 Department of Endocrinology, The Affiliated Lihuili Hospital of Ningbo University, Ningbo, China; 2 Ningbo Institute of lnnovation for Combined Medicine and Engineering, The Affiliated Lihuili Hospital of Ningbo University, Ningbo, China; 3 Department of Geriatrics, Hangzhou First People's Hospital, Hangzhou, China; 4 Ningbo Municipal Center for Disease Control and Prevention, Ningbo, China; 5 Department of Geriatrics, The Affiliated Lihuili Hospital of Ningbo University, No. 57 Xingning Road, Ningbo, China, 86 15257499611

**Keywords:** aging, cognitive function, China Health and Retirement Longitudinal Study, longitudinal studies, sarcopenia

## Abstract

**Background:**

Baseline sarcopenia has been linked to cognitive decline in older adults; however, the impact of longitudinal changes in sarcopenia status on cognitive trajectories remains unclear.

**Objective:**

This aims to examine the association between 2-year transitions in sarcopenia status and subsequent 5-year cognitive trajectories among middle-aged and older adults in China.

**Methods:**

We analyzed data from 8189 participants (median age 58, IQR y; n=432952.9% female) in the China Health and Retirement Longitudinal Study. Sarcopenia status was determined in 2011 and 2013 according to the 2019 Asian Working Group for Sarcopenia criteria, and participants were classified into 7 transition groups based on status changes. Cognitive function was assessed from 2013 to 2018 using standardized *z* scores for executive function and episodic memory. Linear mixed-effects models were applied to evaluate associations between sarcopenia transitions and cognitive trajectories, adjusting for demographic, lifestyle, and health-related covariates.

**Results:**

Progression from a nonsarcopenic state was associated with greater cognitive decline compared to stable nonsarcopenia (*β*=–0.016, 95% CI –0.026 to –0.007; *P*<.001), with greater decline observed among those progressing from possible sarcopenia to sarcopenia (*β*=–0.027, 95% CI –0.054 to –0.001; *P*=.04). In contrast, regression from possible sarcopenia was associated with improved cognitive performance (*β*=0.028, 95% CI 0.015-0.041; *P*<.001). No significant improvement was observed among individuals regressing from established sarcopenia. Subgroup analyses showed consistent benefits of regression from possible sarcopenia across sex, age, residence, and education groups, except among urban residents (*P*=.05).

**Conclusions:**

Progression in sarcopenia status was independently associated with accelerated cognitive decline, whereas regression from possible sarcopenia predicted cognitive benefit. These findings highlight possible sarcopenia as a clinically actionable and potentially reversible stage and underscore the importance of early identification and intervention to preserve cognitive health in aging populations.

## Introduction

Dementia currently affects more than 50 million people worldwide, and its prevalence is projected to reach 150 million by 2050 as the global population continues to age [1]. The societal and economic consequences are profound, with cumulative costs estimated to exceed US $14.5 trillion between 2020 and 2050 [2,3]. Despite intensive research efforts, current therapeutic options remain limited in effectiveness, reinforcing the urgent need to identify modifiable risk factors that may help delay or prevent cognitive decline [4].

Sarcopenia, a progressive skeletal muscle disorder characterized by decline in muscle mass, strength, and physical performance, is highly prevalent among older adults, affecting approximately 10%-27% of this population globally [5]. In addition to its established associations with frailty, falls, disability, and mortality [6-8], emerging evidence suggests a link between sarcopenia and neurocognitive impairment, including mild cognitive impairment and dementia [9-12]. Importantly, unlike many neurodegenerative conditions, sarcopenia is potentially modifiable through targeted interventions such as resistance exercise, adequate protein intake, and nutritional supplementation, making it a promising intervention target for preserving cognitive health in aging populations [13-15].

However, most previous studies have focused on sarcopenia assessed at a single time point and have not accounted for its dynamic nature [9-12]. Sarcopenia may progress or regress over time due to changes in lifestyle or health status, and these transitions may differentially influence cognitive aging. Understanding the longitudinal impact of sarcopenia progression or recovery on cognitive trajectories may provide critical insights into optimal timing of interventions.

To address this gap, we analyzed data from the China Health and Retirement Longitudinal Study (CHARLS), a nationally representative cohort of middle-aged and older adults in China. We investigated whether 2-year transitions in sarcopenia status were associated with subsequent 5-year cognitive trajectories. To our knowledge, this is among the first large-scale longitudinal studies to evaluate the cognitive implications of dynamic changes in sarcopenia status, capturing both progression and regression patterns. We hypothesized that progression to possible sarcopenia or sarcopenia would be associated with accelerated cognitive decline, whereas regression from these states would be linked to a slower rate of cognitive deterioration. Additionally, we examined whether these associations differed across subgroups defined by sex, age, and residential status, and educational attainment, as these factors influence sarcopenia risk [16-18] and modify its relationship with cognitive function.

## Methods

### Study Population

This study used data from the CHARLS, a nationally representative cohort of Chinese adults aged ≥45 years [19]. The CHARLS adopted a multistage, stratified probability sampling strategy, with baseline interviews conducted in 2011 (wave 1) and follow-up assessments in 2013, 2015, and 2018 (waves 2‐4). Sarcopenia status was evaluated in waves 1 and 2, while cognitive function was assessed from Wave 2 onward. Data from wave 5 (2020) were used only in sensitivity analyses due to the partial use of remote video interviews during the COVID-19 pandemic.

Of the 17,708 participants at baseline, we excluded those <45 years of age, those with missing sarcopenia or cognitive data, and those who reported psychiatric or memory-related disorders. Participants lacking sarcopenia assessments in wave 2 or without any cognitive follow-up data were further excluded [20,21].

### Assessment of Sarcopenia

Sarcopenia was defined according to the 2019 criteria of the Asian Working Group for Sarcopenia [22]. Severe sarcopenia was classified as low muscle mass accompanied by both low muscle strength and poor physical performance. Sarcopenia was defined as low muscle mass combined with either low muscle strength or poor physical performance, whereas possible sarcopenia was defined as low muscle strength or reduced physical performance in the absence of low muscle mass.

Appendicular skeletal muscle mass was estimated using an anthropometric prediction equation validated against dual-energy X-ray absorptiometry (DXA), demonstrating high correlation (*R*²=0.90) [23,24]. Sex-specific thresholds for low appendicular skeletal muscle mass or height squared were defined as the lowest 20% of the sex-specific distribution: <7.00 and <7.04 kg/m² for men and <5.25 and <5.32 kg/m² for women in waves 1 and 2, respectively [24,25]. Muscle strength was assessed by handgrip strength, measured twice for each hand using a handheld dynamometer; the maximum value was used for analysis. Low muscle strength was defined as <28 kg for men and <18 kg for women. Physical performance was evaluated using the 5-time chair stand test (≥12 s), walking speed (<1.0 m/s), or the Short Physical Performance Battery (≤9 points) [26].

### Cognitive Function Assessment

Cognitive function was evaluated using structured face-to-face interviews covering 2 cognitive domains: executive function and episodic memory [27,28]. Executive function was assessed through 3 tasks: orientation to time (0‐5 points), serial subtraction (0‐5 points), and figure drawing (0‐1 point), with a total score ranging from 0 to 11. Episodic memory was measured using immediate and delayed recall of a 10-word list, and the mean number of correctly recalled words across both trials was used as the final score (range: 0‐10). Global cognitive function was calculated as the sum of executive function and episodic memory scores, yielding a total score from 0 to 21. All cognitive outcomes were standardized to *z* scores using the baseline mean and standard deviation, with higher values indicating better cognitive performance.

### Covariates

Covariates were selected a priori based on previous research [29]. Demographic factors included age and sex. Socioeconomic variables encompassed education level (elementary or lower vs secondary or higher), marital status (married or partnered vs other), and place of residence (urban vs rural). Health behaviors included smoking status (ever vs never), alcohol use (ever vs never), and BMI (kg/m²). Chronic conditions included self-reported or medication-confirmed diagnoses of hypertension, diabetes, dyslipidemia, heart disease, and stroke.

Pain and depressive symptoms were also included as covariates due to their known associations with both sarcopenia and cognitive outcomes [30-33]. Pain was assessed dichotomously (yes or no). Depressive symptoms were measured using the 10-item Center for Epidemiological Studies Depression Scale (CES-D-10), a validated instrument in the CHARLS population that is widely used in aging research [34,35]. Scores on the CES-D-10 range from 0 to 30, with higher scores indicating more severe depressive symptoms; continuous CES-D-10 scores were included in all models.

### Ethical Considerations

The study protocol of the CHARLS was reviewed and approved by the Biomedical Ethics Review Committee of Peking University (approval No. IRB00001052-11015). All study procedures involving human participants adhered to the ethical standards of Peking University and the Declaration of Helsinki (1964) and its later amendments. Written informed consent was obtained from all participants prior to data collection. All data used in this secondary analysis were deidentified and publicly available; thus, no additional institutional review was required.

### Statistical Analysis

#### Baseline Characteristics

Participants were categorized into groups based on changes in sarcopenia status from wave 1 to wave 2. Baseline characteristics were summarized using medians and IQRs for continuous variables and frequencies with percentages for categorical variables.

#### Sarcopenia Transitions and Cognitive Decline

The primary objective of this analysis was to estimate the rate of cognitive decline associated with different transition patterns in sarcopenia status. Linear mixed-effects models (LMMs) with random intercepts and random slopes were used to model individual cognitive trajectories over time. LMMs account for within-person correlation of repeated measures, accommodate participants with unequal numbers of observations, and are robust to unbalanced longitudinal data under the missing-at-random assumption. Cognitive decline was defined as the annual rate of change in standardized cognitive *z* scores over time, as estimated from the time effect in the LMMs. More negative regression coefficients (*β*) indicated a faster decline in cognitive function. This modeling approach has been widely applied in longitudinal studies of cognitive aging [36-39]. Given the robustness of LMMs to missing outcome data, no imputation procedure was implemented [40,41]. Model estimates were reported as *β* coefficients with 95% CIs.

Models were built sequentially. Model 1 adjusted for age, age squared (to capture nonlinearity), and sex. Model 2 further adjusted for education, marital status, residence, smoking, alcohol use, BMI, and BMI squared. Model 3 additionally controlled for chronic conditions.

#### Subgroup Analyses

Subgroup analyses were stratified by sex (male vs female), age group (<65 vs ≥65 y), residence (urban vs rural), and educational level (elementary or lower vs secondary or higher). These analyses were implemented using 3-way interaction terms between sarcopenia transition group, time, and each subgroup variable.

The sarcopenia transition variable included 3 categories to reflect changes in sarcopenia status between 2 survey waves: (1) progression from nonsarcopenia, (2) change in possible sarcopenia (including progression to sarcopenia and regression to nonsarcopenia), and (3) regression from sarcopenia.

Each subgroup variable was entered into the LMMs as a fixed effect together with its interaction terms with group and time. Effect modification was assessed using the overall *P* value for the 3-way interaction. Stratified associations within each subgroup were further reported to illustrate the pattern and magnitude of associations.

#### Sensitivity Analyses

Several sensitivity analyses were performed to assess the robustness of the findings. First, memory scores from wave 4 were harmonized using equipercentile equating to address potential inconsistencies arising from differences in test versions [42]. Second, a 9-category classification of sarcopenia transitions was applied to capture finer distinctions in status changes and assess whether more granular classification would yield similar associations with cognitive outcomes. Third, wave 5 cognitive data were included, despite partial implementation of remote video assessments during the COVID-19 pandemic, to incorporate the most recent follow-up and examine the stability of the results.

#### Additional Analyses

We further examined whether baseline sarcopenia status (wave 1) predicted subsequent cognitive decline during follow-up. In addition, to identify which diagnostic component contributed most to cognitive deterioration, we evaluated the unique and longitudinal effects of each sarcopenia component—low muscle strength, low muscle mass, and low physical performance—within the fully adjusted LMM (Model 3). For each component, we estimated the time × component interaction term, where a significant coefficient indicated that the component was associated with the rate of cognitive decline over time. We also calculated the semi-partial *R*² (part *R*²) values and corresponding 95% CIs using 1000 bootstrap resamples, representing the proportion of variance in cognitive decline uniquely explained by each component after accounting for covariates.

All analyses were conducted using the R software (version 4.4.1; R Foundation for Statistical Computing), and part *R*² values were obtained with the *partR2* package (0.9.2). A 2-sided *P* value <.05 was considered statistically significant.

## Results

### Participant Grouping and Baseline Characteristics

**Figure 1.**
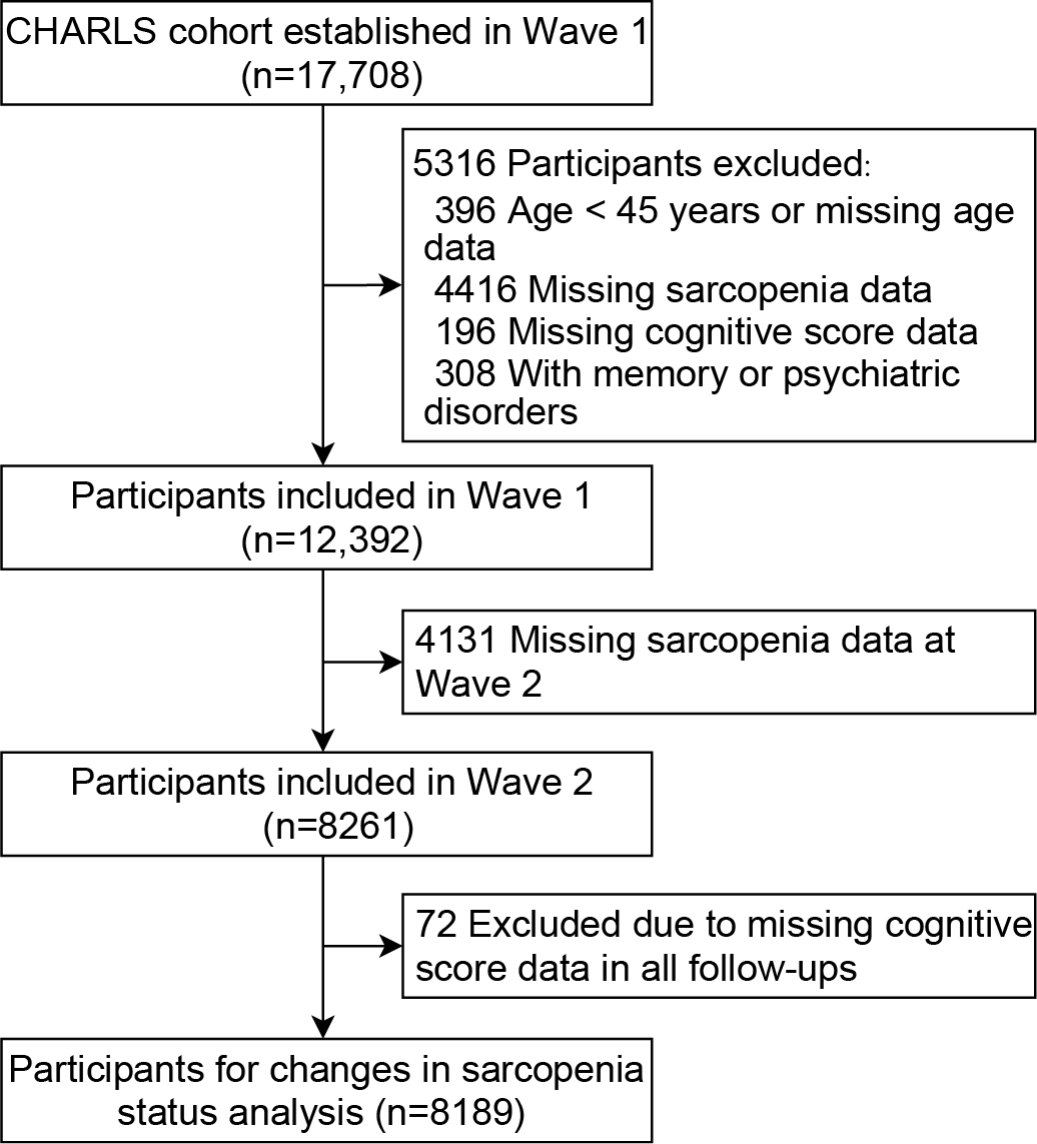
Flowchart illustrating participant selection and inclusion criteria. CHARLS: China Health and Retirement Longitudinal Study.

A total of 8189 participants were included in the final analysis after applying the exclusion criteria (median age 58 y; n=4329, 52.9% female; Figure 1). Participants were initially classified into 9 groups based on sarcopenia status transitions between wave 1 and wave 2 (Table 1).

**Table 1.** Transition of sarcopenia status across wave 1 and wave 2^a^.

Waves 1 and 2		Participants, n (% of total)	Trend of change
Nonsarcopenia (n=4870)			
Nonsarcopenia		3755 (45.9)	Stable
Possible sarcopenia		830 (10.1)	Progression
Sarcopenia		285 (3.5)	Progression
Possible sarcopenia (n=2010)			
Nonsarcopenia		1022 (12.5)	Regression
Possible sarcopenia		839 (10.2)	Stable
Sarcopenia		149 (1.8)	Progression
Sarcopenia (n=1309)			
Nonsarcopenia		161 (2.0)	Regression
Possible sarcopenia		96 (1.2)	Regression
Sarcopenia		1052 (12.8)	Stable

a The time interval between wave 1 and wave 2 survey was 2 years in the China Health and Retirement Longitudinal Study (CHARLS).

However, several transition groups—such as progression from nonsarcopenia to sarcopenia—comprised fewer than 3.5% (n=285) of the total sample, resulting in limited statistical power and unstable model estimates. To improve statistical robustness and model stability, transition groups with similar clinical patterns were consolidated. The final analysis included 7 groups: stable nonsarcopenia, progression from nonsarcopenia, stable possible sarcopenia, regression from possible sarcopenia, progression from possible sarcopenia, stable sarcopenia, and regression from sarcopenia. Baseline characteristics across the 7 transition groups are summarized in Table 2.

**Table 2.** Baseline characteristics of participants by changes in sarcopenia status.

Baseline	Nonsarcopenia		Possible sarcopenia			Sarcopenia	
Trend of change	Stable	Progression	Regression	Stable	Progression	Stable	Regression
Participants, n	3755	1115	1022	839	149	1052	257
Age (y), median (IQR)	55 (49-60)	59 (53-64)	57 (51-63)	62 (56-69)	66 (61-73)	69 (64-75)	64 (60-70)
Sex, n (%)							
Male	1908 (50.8)	527 (47.3)	403 (39.4)	319 (38.0)	67 (45.0)	495 (47.1)	141 (54.9)
Female	1847 (49.2)	588 (52.7)	619 (60.6)	520 (62.0)	82 (55.0)	557 (52.9)	116 (45.1)
Marital status, n (%)							
Married or partnered	3505 (93.3)	986 (88.4)	922 (90.2)	705 (84.0)	117 (78.5)	788 (74.9)	210 (81.7)
Other marital status	250 (6.7)	129 (11.6)	100 (9.8)	134 (16.0)	32 (21.5)	264 (25.1)	47 (18.3)
Education, n (%)^a^							
Elementary or below	2168 (57.7)	836 (75.0)	739 (72.3)	681 (81.2)	139 (93.3)	944 (89.8)	216 (84.0)
Secondary or above	1587 (42.3)	279 (25.0)	283 (27.7)	158 (18.8)	10 (6.7)	107 (10.2)	41 (16.0)
Residence, n (%)							
Rural	2289 (61.0)	767 (68.8)	694 (67.9)	562 (67.0)	102 (68.5)	822 (78.1)	206 (80.2)
Urban	1466 (39.0)	348 (31.2)	328 (32.1)	277 (33.0)	47 (31.5)	230 (21.9)	51 (19.8)
Smoking status, n (%)							
Never smokers	2252 (60.0)	683 (61.3)	669 (65.5)	560 (66.7)	94 (63.1)	597 (56.7)	133 (51.8)
Ever smokers	1503 (40.0)	432 (38.7)	353 (34.5)	279 (33.3)	55 (36.9)	455 (43.3)	124 (48.2)
Drinking status, n (%)							
Never drinkers	2330 (62.1)	764 (68.5)	726 (71.0)	655 (78.1)	116 (77.9)	739 (70.2)	169 (65.8)
Ever drinkers	1425 (37.9)	351 (31.5)	296 (29.0)	184 (21.9)	33 (22.1)	313 (29.8)	88 (34.2)
Hypertension, n (%)							
No	2407 (64.1)	665 (59.6)	608 (59.5)	350 (41.7)	88 (59.1)	648 (61.6)	164 (63.8)
Yes	1348 (35.9)	450 (40.4)	414 (40.5)	489 (58.3)	61 (40.9)	404 (38.4)	93 (36.2)
Dyslipidemia, n (%)^a^							
No	2480 (66.3)	790 (70.9)	668 (65.7)	522 (62.5)	104 (69.8)	838 (79.9)	189 (73.8)
Yes	1259 (33.7)	324 (29.1)	349 (34.3)	313 (37.5)	45 (30.2)	211 (20.1)	67 (26.2)
Diabetes, n (%)^a^							
No	3300 (88.0)	956 (85.8)	867 (84.9)	691 (82.5)	132 (88.6)	965 (91.9)	228 (89.1)
Yes	451 (12.0)	158 (14.2)	154 (15.1)	147 (17.5)	17 (11.4)	85 (8.1)	28 (10.9)
Heart disease, n (%)^a^							
No	3412 (91.3)	976 (87.9)	890 (87.7)	690 (82.7)	127 (85.8)	931 (89.1)	228 (89.1)
Yes	324 (8.7)	134 (12.1)	125 (12.3)	144 (17.3)	21 (14.2)	114 (10.9)	28 (10.9)
Stroke, n (%)^a^							
No	3706 (99.0)	1102 (98.9)	1006 (98.6)	798 (95.6)	146 (98.0)	1032 (98.4)	251 (98.0)
Yes	39 (1.0)	12 (1.1)	14 (1.4)	37 (4.4)	3 (2.0)	17 (1.6)	5 (2.0)
Pain, n (%)^a^							
No	2784 (74.2)	732 (65.7)	620 (60.7)	464 (55.4)	86 (57.7)	646 (61.5)	158 (61.5)
Yes	970 (25.8)	382 (34.3)	402 (39.3)	374 (44.6)	63 (42.3)	405 (38.5)	99 (38.5)
Depressive symptoms^a^^,^^b^, median (IQR)	16 (13-20)	17 (13-22)	18 (14-23)	19 (15-25)	21 (16-25)	19 (15-25)	19 (15-24)
BMI (kg/m^2^)^a^, median (IQR)	23.8 (21.9-26.1)	22.9 (20.9-25.5)	24.1 (22.3-26.4)	24.8 (22.7-27.4)	21.8 (21.0-22.9)	19.1 (17.9-20.1)	19.6 (18.6-20.2)

a Missing data were observed for education (n=1), pain (n=4), diabetes (n=10), stroke (n=21), dyslipidemia (n=30), BMI (n=39), heart disease (n=45), and depressive symptoms (n=369).

b Depressive symptoms were assessed using the 10-item Center for Epidemiological Studies Depression Scale.

### Sarcopenia Transitions and Cognitive Decline

In the fully adjusted model (Model 3), progression from nonsarcopenia was associated with a significantly faster decline in global cognitive function (*β*=–0.016, 95% CI –0.026 to –0.007; *P*<.001), as was progression from possible sarcopenia (*β*=–0.027, 95% CI –0.054 to –0.001; *P*=.04), compared with stable trajectories. In contrast, regression from possible sarcopenia was linked to improvement in global cognition (*β*=0.028, 95% CI 0.01-0.041; *P*<.001). Regression from sarcopenia did not confer a statistically significant cognitive benefit (Table 3).

**Table 3.** Associations between transitions in sarcopenia status and global cognitive decline^e^.

Groups	Model 1^a^		Model 2^b^		Model 3^c^	
	*β*^d^ (95% CI)	*P* value	*β* (95% CI)	*P* value	*β* (95% CI)	*P* value
Nonsarcopenia						
Stable	0 (reference)	—	0 (reference)	—	0 (reference)	—
Progression	–0.016 (–0.025 to –0.007)	<.001	–0.016 (–0.025 to –0.007)	<.001	–0.016 (–0.026 to –0.007)	<.001
Possible sarcopenia						
Stable	0 (reference)	—	0 (reference)	—	0 (reference)	—
Regression	0.027 (0.014 to 0.039)	<.001	0.026 (0.014 to 0.039)	<.001	0.028 (0.015 to 0.041)	<.001
Progression	–0.025 (–0.050 to 0)	.05	–0.028 (–0.053 to –0.003)	.03	–0.027 (–0.054 to –0.001)	.04
Sarcopenia						
Stable	0 (reference)	—	0 (reference)	—	0 (reference)	—
Regression	0.006 (–0.013 to 0.024)	.56	0.006 (–0.013 to 0.025)	.53	0.003 (–0.016 to 0.022)	.75

a Model 1 was adjusted for baseline age, age squared, and sex.

b Model 2 further adjusted for educational level, marital status, residence, smoking status, alcohol consumption, BMI, and BMI squared.

c Model 3 additionally included dyslipidemia, diabetes, hypertension, cardiovascular disease, stroke, depressive symptoms, and pain.

d β: regression coefficient.

e Data were analyzed using linear mixed-effects regression models. The *β* values (95% CI) represent the annual change in cognitive *z* scores (SD/year), relative to the reference group.

Domain-specific analyses yielded results consistent with the findings for global cognition. Progression from nonsarcopenia was associated with declines in executive function (*β*=–0.009, 95% CI –0.018 to –0.001; *P*=.03) and episodic memory (*β*=–0.021, 95% CI –0.034 to –0.009; *P*<.001). Regression from possible sarcopenia was associated with improvements in both executive function (*β*=0.014, 95% CI 0.001-0.026; *P*=.03) and episodic memory (*β*=0.039, 95% CI 0.022-0.056; *P*<.001). No significant improvements were found among those who regressed from sarcopenia (Tables S1–S2 in Multimedia Appendix 1).

### Subgroup Analyses

**Figure 2.**
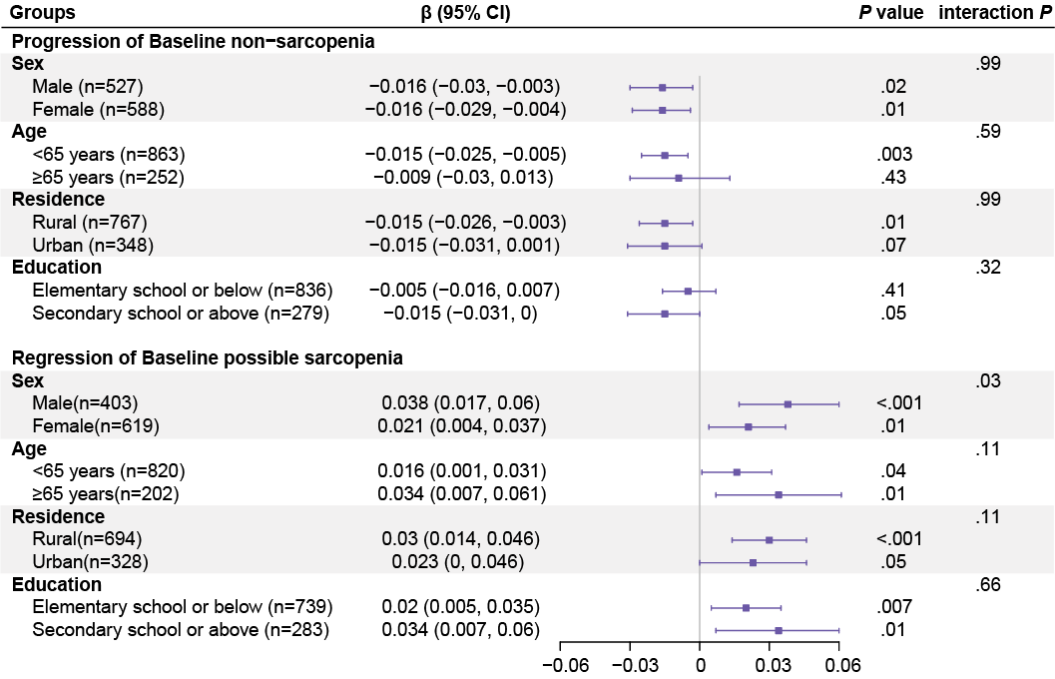
Subgroup analyses of the association between sarcopenia status transitions and global cognitive decline. Results are presented for 2 key transitions: progression from nonsarcopenia and regression from possible sarcopenia. Associations were estimated using linear mixed-effects models, stratified by sex, age group (<65 vs ≥65 y), residential location (urban vs rural), and educational level. Models were adjusted for baseline age and age squared, sex (except in sex-stratified models), education (except in education-stratified models), marital status, residence (except in residence-stratified models), smoking status, alcohol use, BMI and BMI squared, dyslipidemia, diabetes, hypertension, cardiovascular disease, stroke, depressive symptoms, and pain. Error bars indicate 95% CIs. Because transitions from baseline possible sarcopenia include both progression to sarcopenia and regression to nonsarcopenia, a single omnibus interaction *P* value was calculated across these transitions; therefore, the same interaction *P* value is shown here and in Figure S1 in Multimedia Appendix 1.

The results of subgroup analyses for global cognitive function are presented in Figure 2 and Figure S1 in Multimedia Appendix 1. Among participants without sarcopenia at baseline, those who progressed to possible sarcopenia or sarcopenia showed faster declines in cognition compared with those who remained nonsarcopenic. These associations were statistically significant in both men and women, in adults aged <65 years, and among those living in rural areas. Marginal associations were observed among urban residents (*P*=.07) and participants with secondary school education or higher (*P*=.05). However, no significant effect modification was observed across sex, age, residence, or education (all *P* for interaction >.05).

Among participants with baseline possible sarcopenia, transitions demonstrated opposite associations with cognition. Regression to nonsarcopenia was consistently associated with slower cognitive decline across all subgroups, with significant associations observed in most subgroups (all *P*<.05), except for a marginal association among urban residents (*P*=.05). Conversely, progression from possible sarcopenia to sarcopenia was associated with faster cognitive decline, and a significant interaction with sex was observed (*P* for interaction=.03). Specifically, in the progression from possible sarcopenia to sarcopenia, cognitive decline was significant among men (*β*=–0.054, 95% CI –0.094 to –0.014; *P*=.008) but not among women (*β*=–0.004, 95% CI –0.039 to 0.031; *P*=.81).

Among participants with baseline sarcopenia, regression to possible sarcopenia or nonsarcopenia was not associated with significant cognitive benefit in any subgroup (all *P*>.05). Domain-specific subgroup analyses for executive function and episodic memory are presented in Tables S3-S4 in Multimedia Appendix 1.

### Sensitivity Analyses

The findings were generally robust across all sensitivity analyses. When wave 4 memory scores were harmonized using equipercentile equating (Table S5 in Multimedia Appendix 1), the direction of associations remained unchanged; however, among participants who progressed from possible sarcopenia to sarcopenia, effect estimates were attenuated and no longer statistically significant. When analyses were repeated using the original 9-category classification of sarcopenia transitions (Tables S6-S7 in Multimedia Appendix 1), the results were broadly consistent with the primary analysis. The strongest associations were observed among participants progressing from nonsarcopenia to possible sarcopenia (*β*=–0.018, 95% CI –0.028 to –0.007; *P*<.001) and those regressing from possible sarcopenia to nonsarcopenia (*β*=0.028, 95% CI 0.015-0.041; *P*<.001). Incorporating wave 5 cognitive data (Table S8 in Multimedia Appendix 1) did not materially alter the associations, and effect estimates were similar in magnitude and direction to those observed in the main models. Overall, across all sensitivity analyses, progression from nonsarcopenia consistently predicted accelerated global cognitive decline, regression from possible sarcopenia was associated with slower decline, and regression from established sarcopenia did not yield significant cognitive benefit.

### Additional Analyses

We evaluated whether baseline sarcopenia status predicted subsequent cognitive decline. The participant selection flowchart is presented in Figure S2 in Multimedia Appendix 2, and baseline characteristics stratified by sarcopenia status are summarized in Table S9 in Multimedia Appendix 1. As shown in Table S10 in Multimedia Appendix 1, baseline sarcopenia was associated with faster decline in global cognition, executive function, and episodic memory.

We further investigated the relative contributions of individual diagnostic components of sarcopenia to cognitive decline. Longitudinal associations for each component are reported in Table S11 in Multimedia Appendix 1. Among the 3 components, low muscle strength was significantly associated with accelerated cognitive decline (*β*=–0.016, 95% CI –0.027 to –0.004; *P*=.006) and accounted for the largest proportion of explained variance (part *R*²=0.002, 95% CI 0.000‐0.022), as shown in Table S12 in Multimedia Appendix 1. In contrast, low muscle mass was not significantly associated with cognitive decline (*β*=–0.028; *P*=.23), and low physical performance demonstrated only a marginal association (*β*=–0.020; *P*=.06).

## Discussion

This study used longitudinal data from a nationally representative cohort to examine the association between changes in sarcopenia status and cognitive trajectories among middle-aged and older adults. We found that progression from a nonsarcopenic state was significantly associated with accelerated cognitive decline, whereas regression from possible sarcopenia—but not from established sarcopenia—was linked to better cognitive performance. Importantly, the beneficial association of regression from possible sarcopenia with global cognition was consistently observed across multiple sensitivity and subgroup analyses. These findings highlight the importance of early detection and timely intervention during the possible sarcopenia stage, when the potential to preserve cognitive function and delay neurodegeneration may be greatest.

Previous CHARLS-based studies have reported associations between baseline sarcopenia and subsequent cognitive trajectories [11,35]. Our study extends this evidence by focusing on longitudinal changes in sarcopenia status (progression and regression), rather than baseline sarcopenia alone, using nationally representative data. In our cohort, 31.1% (n=2543) of the participants experienced a change in sarcopenia status over 2 years—15.4% (n=1264) progressed and 15.6% (n=1279) regressed—consistent with prior CHARLS findings [24]. Both progression from nonsarcopenia and progression from possible sarcopenia were consistently associated with steeper declines in global cognition, executive function, and episodic memory. In contrast, regression from possible sarcopenia was associated with improved cognitive outcomes. Notably, regression from established sarcopenia was not associated with meaningful cognitive gains, suggesting a limited therapeutic window once sarcopenia becomes entrenched.

In the subgroup analyses, participants who progressed to possible sarcopenia or sarcopenia exhibited faster cognitive decline than those who maintained a stable nonsarcopenic state. Significant associations were observed in certain subgroups (eg, men, women, individuals aged <65 y, and rural residents), whereas other subgroups showed attenuated or nonsignificant associations. None of the interaction terms for sex, age, residence, or education reached statistical significance. Consistent with guidance from Sun et al [43], the nonsignificant interactions observed in our study do not imply identical effects across subgroups but may reflect small true differences that our sample lacked adequate power to detect. Although the direction of associations between sarcopenia progression and cognitive decline was consistent across demographic strata, subtle population heterogeneity cannot be excluded. These findings underscore the need for larger, adequately powered prospective studies to validate potential subgroup-specific effects.

For instance, in the age-stratified analyses, the association between sarcopenia progression and accelerated cognitive decline was evident among participants aged <65 years but attenuated in those aged ≥65 years, although the interaction effect was not statistically significant. This finding contrasts with prior cross-sectional CHARLS research [44], which reported significant associations between sarcopenia and cognitive function in older adults. Although Du et al [44] examined cross-sectional relationships in participants aged ≥60 years, our longitudinal design captured within-person changes in cognition over time—an approach that better reflects temporal dynamics but is also influenced by attrition and survivorship effects. Selective attrition in longitudinal cohorts often leads to loss of participants with poorer cognitive function or declining health, leaving a more resilient survivor subset with reduced between-person variability [45]. Consequently, those at the greatest risk for cognitive decline may be disproportionately lost to follow-up, attenuating observed effects among older adults. Moreover, unaccounted competing risks such as mortality or severe morbidity may further underestimate associations; Austin and Fine [46] noted that treating participants who experience competing events as censored can bias estimates downward by excluding high-risk individuals. Finally, the smaller sample size in the ≥65-year group likely reduced statistical power and widened confidence intervals. Collectively, these factors likely contributed to the absence of statistically significant associations in older adults despite consistent effect directions across age strata.

Importantly, regression from possible sarcopenia was consistently associated with improved global cognitive function across all subgroups, with significant results (all *P*<.05) except among urban residents, where the association reached marginal significance (*P*=.05). These findings suggest that recovery from possible sarcopenia—representing an intermediate and potentially reversible stage—may provide a critical window for intervention to preserve cognitive health.

Our sensitivity analyses demonstrated that the main findings were robust. Incorporating wave 5 data, which reflect the most recent cognitive assessments, yielded results nearly identical to the primary analyses, underscoring the stability and reproducibility of our findings. In contrast, when wave 4 memory scores were harmonized using equipercentile equating, associations in the progression from possible sarcopenia to sarcopenia group were attenuated and no longer statistically significant. This attenuation likely reflects the conservative nature of the equating method, which reduces variability across test versions and may compress score distributions, thereby lowering statistical power despite consistent effect directions [42].

Similarly, when the original 9-category classification of sarcopenia transitions was used, the association between progression from nonsarcopenia to sarcopenia and cognitive decline became weaker. This was primarily due to the very small number of participants in this transition group, which reduced statistical power and produced less stable estimates. Moreover, the strong association observed in the nonsarcopenia to possible sarcopenia group is biologically plausible. Possible sarcopenia represents an early transitional stage characterized predominantly by reduced muscle strength, a parameter that has been shown to be a more sensitive indicator of functional decline and adverse health outcomes than muscle mass alone. Consistent with our additional analyses, low muscle strength—but not low muscle mass—was independently associated with faster cognitive decline. These findings align with evidence from population-based cohorts and Mendelian randomization studies showing that handgrip strength is more strongly linked to cognitive performance than muscle mass [47,48]. Therefore, cognitive deterioration may become detectable during the possible sarcopenia stage, before the onset of advanced muscle wasting in established sarcopenia. Across all analyses, however, regression from possible sarcopenia consistently demonstrated cognitive benefits, reinforcing the notion that this transitional stage is particularly amenable to intervention [49].

Several biological mechanisms may explain these associations. The muscle-brain axis has been proposed as a key pathway linking musculoskeletal and cognitive health [50]. Skeletal muscle secretes myokines—signaling molecules that regulate systemic inflammation, metabolic homeostasis, and neuroplasticity [51,52]. Sarcopenia may reduce myokine production and disrupt neuroprotective signaling, whereas reversal through physical activity may restore these pathways [52-54]. In addition, sarcopenia progression is associated with chronic inflammation and oxidative stress—both implicated in cognitive decline and neurodegeneration [55-58]—which may be mitigated through improved muscle function [59,60]. Emerging evidence also suggests a role of gut microbiota dysbiosis in sarcopenia-related neuroinflammation, underscoring the potential importance of the gut-muscle-brain axis [61,62].

These findings have important implications for clinical practice and public health. Routine screening for possible sarcopenia should be incorporated into cognitive risk assessment protocols for aging populations. Such screening is feasible using simple measures—such as grip strength and chair-stand tests—that can be implemented in primary care or community settings without specialized equipment. Early detection may help identify individuals at elevated risk of cognitive decline who could benefit from timely, multidisciplinary interventions. In our cohort, more than half of participants with possible sarcopenia reverted to a nonsarcopenic state (Table 1), reinforcing the reversibility of early-stage functional decline and the importance of timely intervention. Growing evidence from intervention trials suggests that resistance training, alone or combined with nutritional supplementation, can improve muscle function and sarcopenia-related outcomes [13-15], supporting the potential reversibility of early-stage sarcopenia. Future studies should evaluate whether such interventions can also yield sustained cognitive benefits.

This study has several limitations. First, its observational nature precludes causal inference. While associations were consistent across sensitivity analyses, randomized controlled trials are needed to establish causality. Second, sarcopenia was assessed using a validated anthropometric prediction equation rather than dual-energy DXA, the reference standard. Although highly correlated with DXA (*R*²=0.90) [23], some misclassification may have occurred. However, the 15% sarcopenia prevalence in our study aligns with previous meta-analyses using DXA or BIA, supporting its validity [5]. Third, modifications to the episodic memory test in 2018 may have introduced measurement inconsistencies; therefore, we applied equipercentile equating to harmonize memory scores across waves. Although this conservative adjustment attenuated associations in the progression from possible sarcopenia to sarcopenia group, the overall direction of effects remained consistent, supporting the robustness of the main findings [42]. Fourth, although physical exercise has been shown to be significantly associated with both cognitive function and sarcopenia [63,64], information on physical activity was missing in 61.0% (n=4995/8189) of participants in our cohort. Therefore, physical exercise was not included as a covariate in our analyses, and residual confounding related to physical activity cannot be excluded. Finally, residual confounding remains possible. Relevant factors such as hearing loss or social isolation—which may influence both sarcopenia and cognitive decline—were not fully captured and should be explored in future research [65].

Progression from a nonsarcopenic state—including progression from possible sarcopenia—is associated with accelerated cognitive decline, whereas regression from possible sarcopenia is linked to cognitive benefit. These findings identify possible sarcopenia as a critical and potentially reversible stage in the trajectory toward sarcopenia and cognitive impairment. They also support incorporating routine screening for possible sarcopenia into cognitive risk assessment protocols for aging populations. Given the simplicity, low cost, and feasibility of screening measures such as grip strength and chair-stand tests, implementation in primary care and community settings is both practical and scalable. Early identification and intervention at the possible sarcopenia stage may offer a key opportunity to preserve cognitive health and delay neurodegenerative processes. Future research should prioritize developing and evaluating targeted, evidence-based strategies to prevent sarcopenia progression and promote cognitive resilience in at-risk populations.

## Supplementary material

10.2196/78277 Multimedia Appendix 1 Associations between sarcopenia transitions and cognitive trajectories; subgroup analyses by demographic factors; three sensitivity analyses; and associations between baseline sarcopenia and cognition, including contributions of muscle strength, mass, and performance.

10.2196/78277 Multimedia Appendix 2 Subgroup analyses of sarcopenia transition effects on global cognition and the participant flow for the baseline sarcopenia analysis.

## Abbreviations

BMI: body mass index
CES-D-10: 10-item Center for Epidemiological Studies Depression Scale
CHARLS: China Health and Retirement Longitudinal Study
DXA: X-ray absorptiometry
LMM: linear mixed-effects model
