# Supplementary material for: Transitions in Sarcopenia Status and Cognitive Trajectories Among Middle-Aged and Older Adults in China: Longitudinal Cohort Study
Source: JMIR Aging. 2025 Dec 16;8:e78277. doi: 10.2196/78277 (PMC12707442; doi:10.2196/78277)
Supplement: Multimedia Appendix 2 [file aging-v8-e78277-s002.docx]

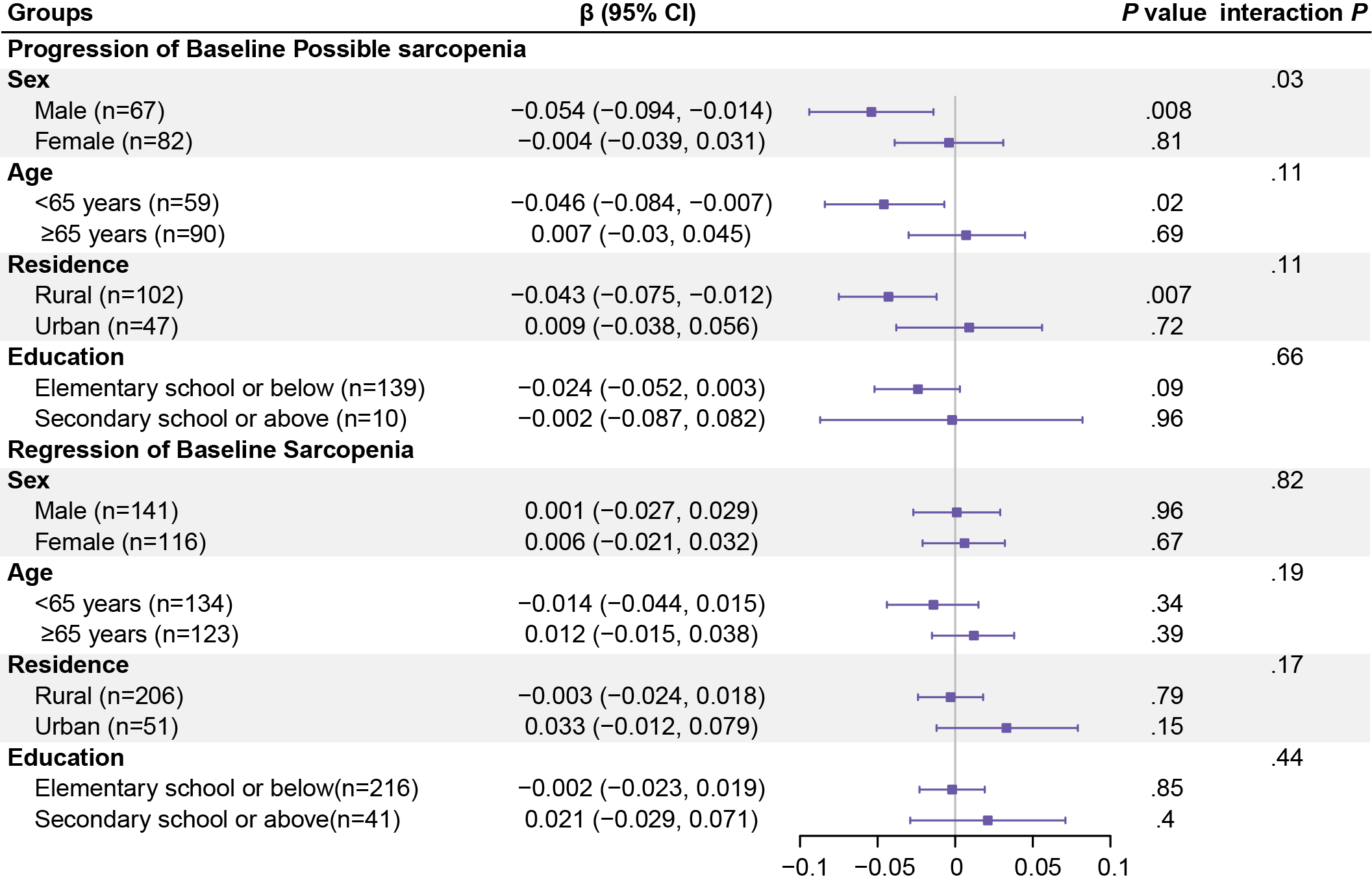


**Figure S1. Subgroup analyses of the association between sarcopenia status transitions (progression from possible sarcopenia and regression from sarcopenia) and global cognitive trajectories.** Associations were estimated using linear mixed-effects models stratified by sex, age group, residential location, and educational level. Models were adjusted for baseline age and age²; sex (except in sex-stratified models); education (except in education-stratified models); marital status; residence (except in residence-stratified models); smoking; alcohol use; BMI and BMI²; dyslipidemia; diabetes; hypertension; cardiovascular disease; stroke; depressive symptoms; and pain. Error bars indicate 95% confidence intervals. Because transitions from baseline possible sarcopenia include both progression to sarcopenia and regression to non-sarcopenia, a single omnibus interaction P value was calculated to test for effect modification across subgroup strata. BMI: body mass index; CI: confidence interval.


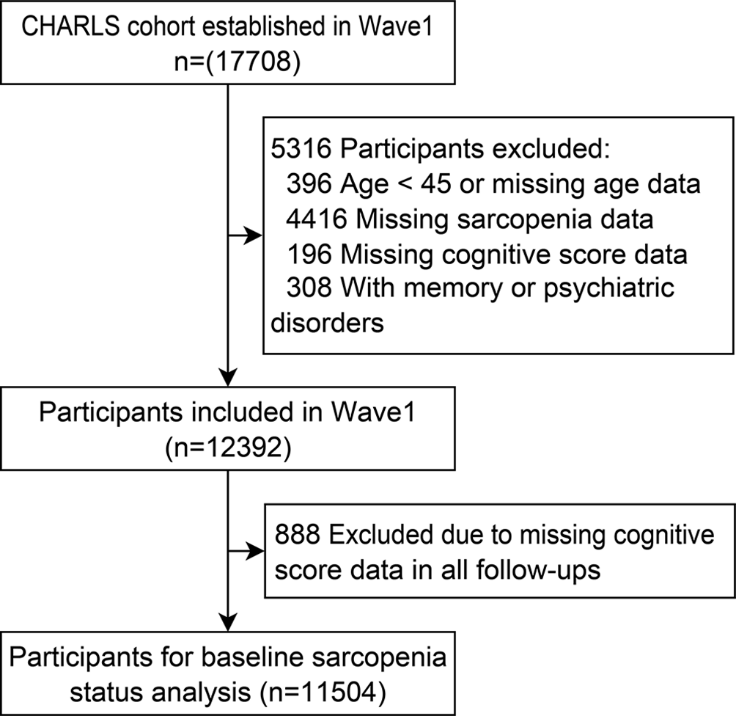


**Figure S2.** Flowchart of participant selection for baseline sarcopenia analysis
